# GISDD: A comprehensive global integrated sequence and genotyping database platform for dengue virus, facilitating a stratified coordinated surveillance strategy

**DOI:** 10.1002/imo2.70048

**Published:** 2025-08-27

**Authors:** Xiang Guo, Pingying Teng, Ziyao Li, Liqiang Li, Xiaohua Liu, Xiaoqing Zhang, Yuji Wang, Minling Hu, Wenwen Ren, Shu Zeng, Haiyang Chen, Liu Ge, Shihan Liu, Zhiqiang Peng, Jiufeng Sun, Xin Zhang, Lei Luo, Jie Peng, Benyun Shi, Rangke Wu, Jiming Liu, Fuchun Zhang, Xiao‐Guang Chen, Tong Chen, Xiaohong Zhou

**Affiliations:** ^1^ Institute of Tropical Medicine, Department of Pathogen Biology, School of Public Health, Southern Medical University; Guangdong Provincial Key Laboratory of Tropical Disease Research; Key Laboratory of Infectious Diseases Research in South China Ministry of Education Guangzhou Guangdong China; ^2^ School of Basic Medical Sciences Henan University Kaifeng China; ^3^ Department of Parasitology Guilin Medical University Guilin China; ^4^ Department of Clinical Laboratory, The Third People's Hospital of Shenzhen, Southern University of Science and Technology, National Clinical Research Center for Infectious Diseases, Guangdong Provincial Clinical Research Center for Infectious Diseases (Tuberculosis) Shenzhen Clinical Research Center for Tuberculosis Shenzhen China; ^5^ Guangdong Provincial Center for Disease Control and Prevention Guangzhou China; ^6^ Guangdong Provincial Institute of Public Health Guangdong Provincial Center for Disease Control and Prevention Guangzhou China; ^7^ Guangzhou Center for Disease Control and Prevention Guangzhou China; ^8^ Department of Infectious Disease, Nanfang Hospital Southern Medical University Guangzhou China; ^9^ College of Computer and Information Engineering Nanjing Tech University Nanjing China; ^10^ The School of Foreign Studies Southern Medical University Guangzhou China; ^11^ Department of Computer Science Hong Kong Baptist University Hong Kong China; ^12^ Guangzhou Medical Research, Institute of Infectious Diseases, Infectious Disease Center, Guangzhou Eighth People's Hospital Guangzhou Medical University Guangzhou China; ^13^ State Key Laboratory for Quality Ensurance and Sustainable Use of Dao‐di Herbs, National Resource Center for Chinese Materia Medica China Academy of Chinese Medical Sciences Beijing China

## Abstract

GISDD, the Global Integrated Sequence and Genotyping Database for Dengue Virus (DENV), provides an integrated online analysis platform encompassing tools such as GISDDrlearn, GISDDprimer, and GISDDref, enabling swift identification and tracing of well‐established DENV genotypes, subgenotypes, and clades, thereby facilitating a stratified global coordinated surveillance strategy.

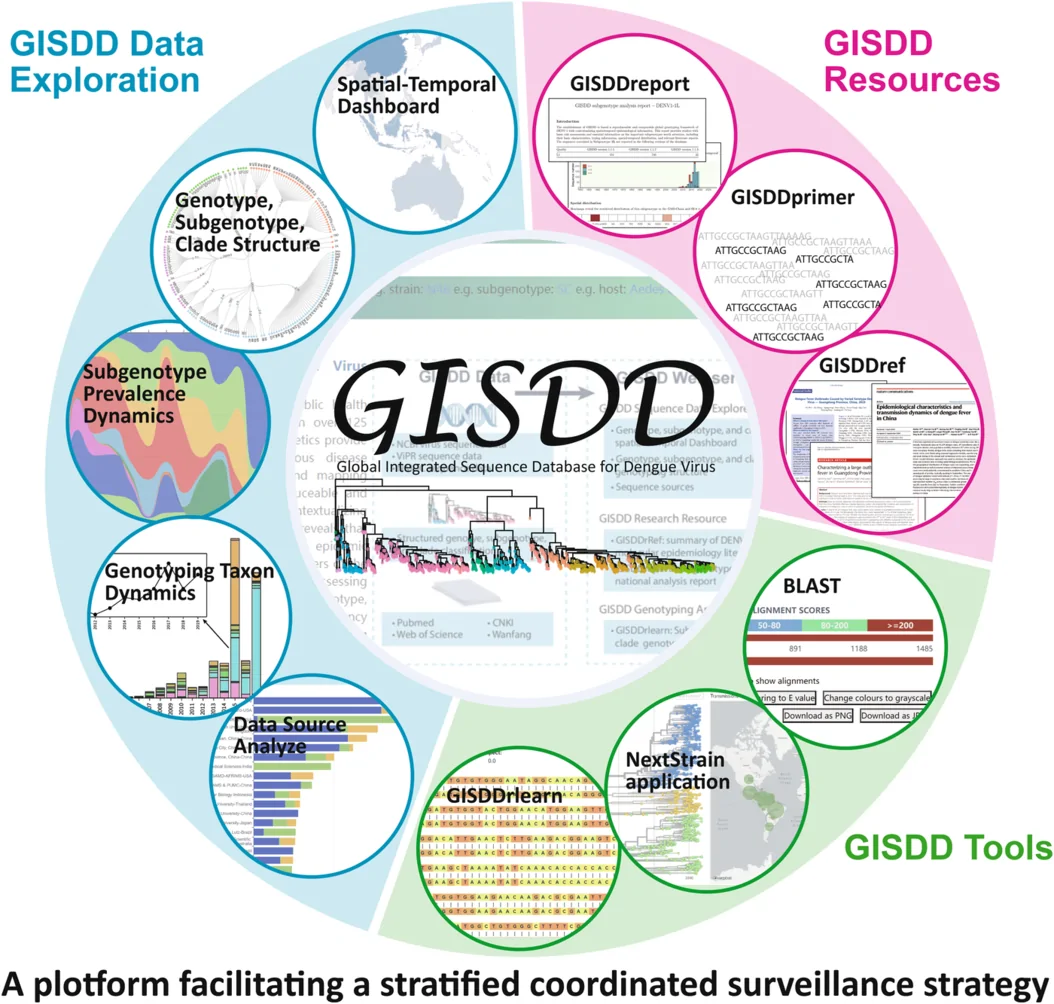

## ETHICS STATEMENT

No animals or humans were involved in this study.

To the Editor,

Dengue virus (DENV), a member of the Flavivirus genus, primarily transmitted by *Aedes egypti* and *Ae. albopictus*, increasingly poses a significant public health challenge globally, endangering half of the world's population across more than 125 countries [1, 2]. The escalating burden of dengue is attributed to various factors, including unplanned urbanization, climate change, international trade, and traffic integration. These factors, together with the rapid spreading of invasive vectors, contribute to its irreversible trend [2, 3]. Recent advances in virus sequencing technologies and phylogenetic analyses have emerged as powerful tools for addressing pivotal questions in viral infectious disease epidemiology [4, 5, 6]. We previously established a reproducible and globally comparable genotyping framework for DENV‐1, that integrates spatiotemporal epidemiological insights [7], and utilized it to elucidate the invading and diffusing patterns of DENV‐1 epidemics in Guangdong, China, through phylodynamics [8]. This framework has identified three key drivers underpinning the rapid dissemination of dengue: the persistence of traditional endemic sources, diffusion from emerging epidemic areas, and the potential presence of concealed epidemics [7].

On the other hand, the exponential growth of data and intricate multi‐source data architectures has facilitated the swift establishment of pertinent genetic information databases [9]. However, accessing public repositories such as GenBank [10] and ViPR [11] renders challenges for researchers seeking specific information like geographical details, host species identification, and travel histories. Additionally, distinguishing non‐outbreak‐related data, including laboratory‐passaged and modified strains, is difficult. The potential to explore “fine‐grained” data from literature and reports remains largely untapped. In the past decades, especially the past 10 years, a series of specialized databases for virology, such as GISAID [12, 13], CoV‐lineages [14], Pathogenwatch [15], and Nextstrain [16], have emerged to enhance the surveillance and genotyping of pathogens. Despite these advancements, a notable gap exists in resources dedicated to DENV. To bridge this gap, we have created the Global Integrated Sequence Database for Dengue viruses (GISDD), freely accessible at https://www.bic.ac.cn/GISDD/, a meticulously curated repository of DENV sequence information, aimed at providing a comprehensive platform for efficient browsing of global three‐level hierarchical DENV genotyping framework.

## 1 FEATURES OF GISDD DATA

**Figure 1:**
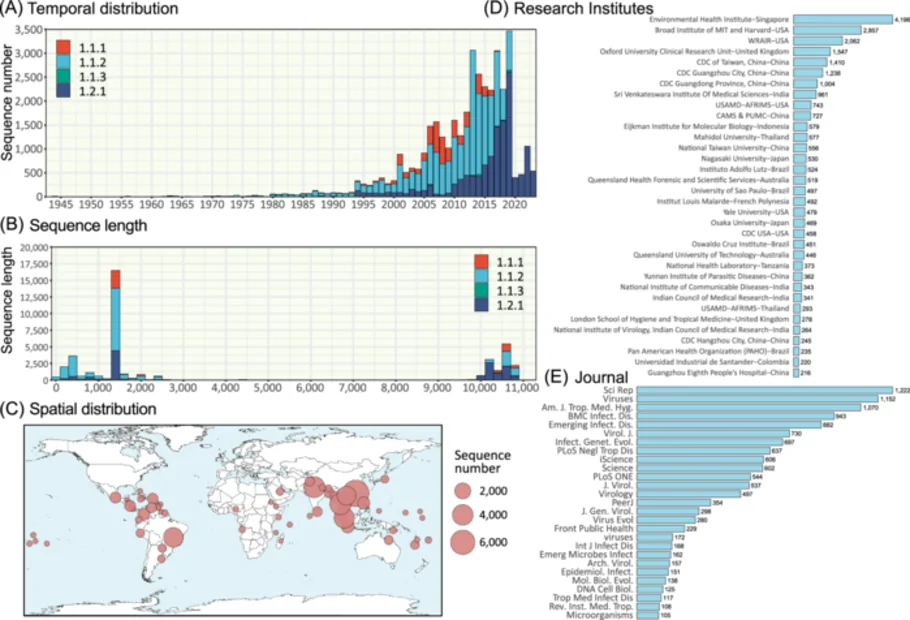
Key features of Global Integrated Sequence Database for Dengue viruses (GISDD). The temporal distribution (A), length distribution (B), and spatial distribution (C) of reported sequences in GSIDD. The top 30 institutions (D), and the top 30 journals (E) ranked by the number of DENV sequences they have reported.

The GISDD (version 1.3.2) has compiled a comprehensive collection of 41,336 sequences, with 16,058 for DENV‐1, 13,923 for DENV‐2, 7579 for DENV‐3, and 3776 for DENV‐4. The temporal distribution of these sequences reported reveals a gradual accumulation over time, peaking around 2013–2019, with approximately 1000 sequences being added annually since 2005 (Figure 1A). In recent years, there has been a decline in the number of sequences, which may be attributed to the duration of ongoing studies and potential delays in data sharing and publication timelines (Figure 1A). The lengths of database sequences cluster within three primary ranges: 200–500 bp, 1300–1500 bp, and 10,400–10,800 bp (Figure 1B), corresponding to commonly targeted genomic regions of DENV, such as the Non‐structure 1(NS1) gene fragment (~240 bp), the full‐length Envelope (E) gene (~1485 bp), and the complete genome (~11 kb). The geographic distribution of database sequences pertaining to DENV exhibits a broad reach, with a pronounced concentration of reported strains originating from East Asia and Southeast Asia, in contrast to the relatively sparse reporting in Africa (Figure 1C). A total of 566 institutions worldwide, including universities, research facilities, and hospitals, have contributed to the documentation of dengue sequences (Figure 1D). These sequences have been featured in 683 published articles across 140 journals (Figure 1E).

## 2 WEB INTERFACE IN GISDD

GISDD provides a user‐friendly web interface which enables users to efficiently query, browse, scrutinize, and explore data within the DENV genotyping framework (Figure S1). The interface features an intuitive navigation bar that guides users to key sections, including “Browse,” “Explore,” “Resources,” “Tools,” and “Workflow.”

## 3 DATA EXPLORATION IN GISDD

To enhance user convenience in querying and browsing data, the GISDD incorporates a dedicated “Browse” page, granting users a direct access to the original data within the database. For a more detailed examination of individual sequences, users can click on a function button (represented by four small squares), enabling them to view comprehensive information and even download all available data with a simple click. For example, the “Spatial‐Temporal Dashboard” is a powerful webpage that allows users to perform a comprehensive analysis of the spatial‐temporal distribution of DENV sequences. By selectively filtering on specific genotypes, subgenotypes, clades, or defining a precise sequence time range, users can create summaries that offer valuable insights into the DENV's temporal and spatial dynamics (Figure S2).

## 4 RESOURCES IN GISDD

The “GISDDreport” website comprises two series of analysis reports meticulously crafted using R Markdown, with a focus on subgenotype/clade and location analyses (Figure S1). One series focuses on subgenotype/clade analysis, providing risk assessments and key information about clades, including their characteristics, genotyping, distribution patterns, and related literature. The other series addresses location analysis, offering risk assessments and details on significant DENV subgenotypes in each epidemic country. To create a thorough overview of dengue molecular epidemiology research and explore pioneering topics, we systematically retrieved 591 studies for inclusion in our descriptive analysis. This compilation is now accessible through GISDDref, which features interactive dashboards that offer an extensive summary of global and Chinese dengue molecular epidemiological researches (Figure S1). GISDDprimer is another web application designed to facilitate the search for both published and novel primer pairs targeting the DENV fragments (Figure S1). The summary table displays detailed information, including primer name, primer pair/set name, primer sequences, primer position within the DENV genome, primer length, targeted serotype, targeted fragment, possible off‐target ratio for all inclusive DENV sequences, potential off‐target sequences categorized by subgenotype, and the associated reference literature.

## 5 WEBSERVER OF TOOLS IN GISDD

The “Tools” page of GISDD is equipped with a suite of web tools, including GISDDrlearn, blast for DENV, and basic phylogeny, tailored to address the rapid growth in genomic data. The exponential expansion in virus genome data production has necessitated the development of innovative analytical methods capable of handling a larger number of genomes than previously accessible. As an important machine learning model, Random Forest comprises an ensemble of decision trees, each constructed using a subset of the training sample. The structural analogy between Random Forest and evolution trees reveals an intriguing connection [14]. Leveraging this concept, we developed GISDDrlearn, a R script application, for assigning DENV sequences to serotype, genotype, subgenotype, and clade based on the Random Forest algorithm (Figure S1). Given the accessibility and affordability of the E gene of DENV, our high‐resolution genotyping with GISDDrlearn can bridge classical E‐gene‐based genotyping methods with the emerging techniques of genomic epidemiology. All GISDDrlearn models, trained with complete E sequences of DENV and their designated serotypes, genotypes, subgenotypes, and clades, can assign 1000 E sequences in approximately 30 s.

## 6 SEQUENCE GENOTYPING‐INTEGRATED THINKING‐RISK ESTIMATION (SIR) WORKFLOW IN GISDD

Integration of phylogenetic analyses of SARS‐CoV‐2 sequences with epidemiological data has significantly enhanced our understanding of cross‐border spatial and temporal dissemination, empowering public health officials and policymakers with crucial evidence to inform effective interventions [4]. However, conducting DENV monitoring and research presents two major challenges. Firstly, although Next‐Generation Sequencing (NGS) has been employed as a surveillance tool in high‐income countries for several years, its application remains uneven across low‐ and middle‐income countries [17]. Barriers to implementing NGS include dependence on external funding, supply chain complexities, a shortage of trained personnel, and limited quality assurance mechanisms [17]. Secondly, the absence of a collaborative organization for data sharing in dengue research hampers timely data synchronization and necessitates future improvements. These challenges frequently complicate the task of tracing the origin of DENV surveillance through phylogenetic analysis, as considerable time lags between the collection of samples and the availability of their associated sequences may result in the prominent absence of certain viral populations or render it infeasible to trace the origin based on evolutionary relationships.

Our previous systematic research findings have laid a solid foundation for risk warning and tracing the import of DENV in global transmission. Initially, we developed a unified global genotyping framework for DENV and identified distinct epidemic patterns in dengue transmission, revealing stratified spatio‐genetic epidemic pairs characterized by Continent‐Genotype, Region‐Subgenotype, and Nation‐Clade distributions [7]. Moreover, in another investigation focusing on the complexity of the population structure of DENV‐1 epidemics in China within the global genotyping framework, we observed a power‐law distribution in the number of sequences belonging to specific clades. Notably, over 80% of these sequences were concentrated within seven particular clades: 1E1, 1L1, 5C1, 1K1, 1L2, 1H4, and 1J7, which were subsequently designated as Clades of Concern (COCs). Through comprehensive analyses that integrated phylogeny with epidemiological information pertaining to the importation sources of COC clusters, we found that the origin of 18.8% to 100% of the transmission clusters within the seven COCs could not be definitively inferred [8].

**Figure 2:**
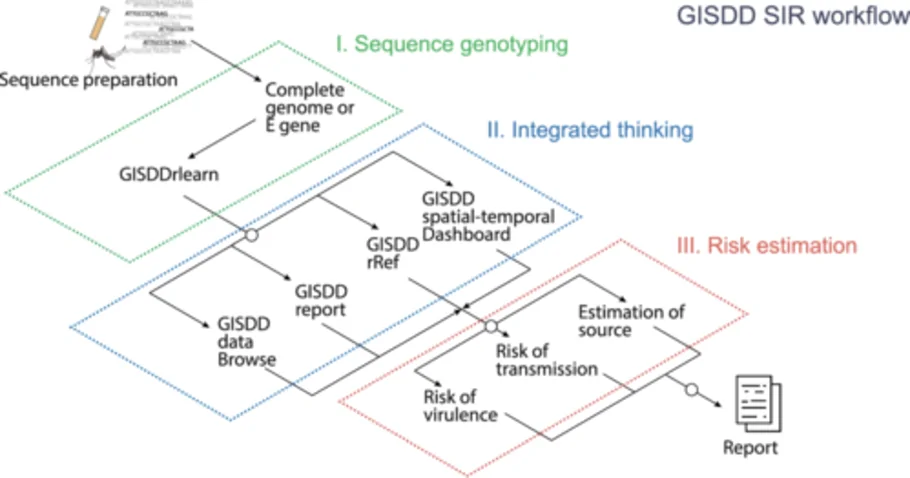
Overview of Global Integrated Sequence Database for Dengue viruses (GISDD) Sequence genotyping‐Integrated thinking‐Risk estimation (SIR) workflow. Upon obtaining sequence data containing complete E protein sequences for the SIR workflow, users need to determine the genotyping of these acquired DENV sequences during the initial stage. Then SIR integrated three key stages: (1) Sequence genotyping, (2) Integrated thinking, and (3) Risk estimation. The workflow offers a user‐friendly web interface for sequence submission and automated report generation within the GISDD platform.

Based on the aforementioned research findings, we propose to implement the SIR workflow for demonstrating DENV transmission dynamics through the GISDD platform, which comprises three distinct stages as depicted in Figure 2. Within this workflow, there are multiple options available for sequence preparation, including the utilization of NGS and other methods. We offer publicly accessible protocol within the GISDD resource. Upon obtaining sequence data containing complete E protein sequences for the SIR workflow, users need to determine the genotyping of these acquired DENV sequences during the initial stage (Figure 2). Users can opt for tools like GISDDrlearn from the GISDD resource to perform genotyping. Rather than solely focusing on the construction of molecular trees for genotyping determination, the SIR workflow incorporates “Integrated Thinking” into its second stage to analyze the subgenotypes and clades of DENV linked to these sequences, by scrutinizing their spatiotemporal distribution and pertinent information extracted from GISDD reports (Figure 2). Phylogeny is also employed as a fundamental component to support comprehensive analysis. In the third stage, risk estimation pertaining to local dengue transmission is carried out based on the aforementioned resources that are specific to the particular clade and subgenotype in question (Figure 2). For this SIR workflow, we provide a template within GISDD, enabling users to directly submit their sequences through a webpage for analysis and receive the corresponding workflow reports.

## 7 CASE 1: COMPREHENSIVE ANALYSIS OF POPULATION STRUCTURE OF DENV‐2 STRAINS CIRCULATING IN CHINA

From a macroscopic perspective, the results obtained from fractal analysis can be utilized for systematic surveillance. We conducted a comprehensive investigation into the prevalence of DENV‐2 in China during recent years. The viral populations in China encompassed 22 subgenotypes. belonging to the globally and widely circulating genotypes III, IV, V, and VI of DENV‐2 (Figure S3A). Notably, among the total DENV‐2 cases in China, the subgenotypes 3AM, 3D, and 5B were found to exceed 10% (Figure S3A). Furthermore, from a temporal dynamic perspective, we observed an increase in the abundance of prevalent DENV‐2 strains in China in recent years (Figure S3B). Specifically, there was a growth trend for subgenotype 3AM. Based on this observation, it can be inferred that post‐2019 onwards, subgenotype 3AM may continue to pose a threat to China (Figure S3B).

## 8 CASE 2: SIR ANALYSIS FOR A LOCAL DENGUE EPIDEMIC IN SHENZHEN, CHINA IN 2024

We conducted a case study on a local epidemic that occurred at a construction site in Bao'an District, Shenzhen, China in 2024. The complete E gene sequences acquired from this outbreak were analyzed using the SIR workflow via the GISDD platform. Subsequently, based on GISDD classification, these viral strains were identified as belonging to DENV‐2 subgenotype 3AM (Figure S3). In recent years, this subgenotype has emerged as the predominant epidemic strain of DENV‐2 globally, accounting for approximately 80% of reported sequences worldwide during that year (Figure S3). Regarding its impact on China, extensive documentation of this subgenotype exists for the years 2015, 2017, and 2019 (Figure S3). From a tracing perspective, it is plausible that this subgenotype originated from Southeast Asian countries such as Malaysia, Singapore, and Indonesia, with potential contributions from countries along the Mekong River, like Cambodia. Based on genetic analysis, the current outbreak is believed to be a locally transmitted epidemic resulting from independent introductions. From a pathogenesis perspective, there is currently a lack of information concerning the transmission or pathogenicity associated with DENV‐2 subgenotype 3AM.

## AUTHOR CONTRIBUTIONS

**Xiang Guo**: Data curation; formal analysis; Writing—original draft. **Pingying Teng**: data curation. **Ziyao Li**: Data curation. **Liqiang Li**: Conceptualization; Writing—original draft. **Xiaohua Liu**: Data curation. **Xiaoqing Zhang**: Data curation. **Yuji Wang**: Data curation. **Minling Hu**: Data curation. **Wenwen Ren**: Data curation. **Shu Zeng**: Data curation. **Haiyang Chen**: Data curation. **Liu Ge**: Data curation. **Shihan Liu**: Formal analysis. **Zhiqiang Peng**: Writing—review and editing. **Jiufeng Sun**: Writing—review and editing. **Xin Zhang**: Writing—review and editing. **Lei Luo**: Writing—review and editing. **Jie Peng**: Writing—review and editing. **Benyun Shi**: Writing—review and editing. **Rangke Wu**: Writing—review and editing. **Jiming Liu**: Writing—review and editing. **Fuchun Zhang**: Conceptualization. **Xiao‐Guang Chen**: Conceptualization. **Tong Chen**: Conceptualization; formal analysis. **Xiaohong Zhou**: Conceptualization; funding acquisition; Writing—review and editing.

## 9 CONFLICT OF INTEREST STATEMENT

The authors declare no conflicts of interest.

## Supporting information


**Figure S1:** Overview of GISDD data sources, web logical architecture, content.
**Figure S2:** The user interface of data exploration in GISDD.
**Figure S3:** SIR workflow implementation two cases.
**Figure S4:** Parameter optimization and classification effect of GISDDrlearn.

**Table S1:** Criteria for the classification of sequence quality levels.
**Table S2:** Version update log of GISDD.
**Table S3:** Confusion matrix for each sequence's serotype, genotype and subgenotype predictions in the training set of GISDDrlearn model.

## Data Availability

The GISDD database is freely available at https://www.bic.ac.cn/GISDD/. The code scripts and raw data of this study are available at https://github.com/GuoXiang9399/GISDDrlearn-training, https://github.com/GuoXiang9399/GISDDrRef, and https://github.com/GuoXiang9399/GISDDrPrimer. Supplementary materials (methods, figures, tables, graphical abstract, slides, videos, Chinese translated version, and update materials) may be found in the online DOI or iMetaOmics http://www.imeta.science/imetaomics/.
