# Supplementary material for: GISDD: A comprehensive global integrated sequence and genotyping database platform for dengue virus, facilitating a stratified coordinated surveillance strategy
Source: IMetaOmics. 2025 Aug 27;2(4):e70048. doi: 10.1002/imo2.70048 (PMC12806018; doi:10.1002/imo2.70048)
Supplement: Supplementary file 1 — Figure S1: Overview of GISDD data sources, web logical architecture, content. Figure S2: The user interface of data exploration in GISDD. Figure S3: SIR workflow implementation two cases. Figure S4: Parameter optimization and classification effect of GISDDrlearn. [file IMO2-2-e70048-s002.docx]

**Supporting Information to:**

**GISDD: a comprehensive global integrated sequence and genotyping database platform for dengue virus, facilitating a stratified coordinated surveillance strategy**

**Running title:** GISDD: a comprehensive global sequence and genotyping database platform for dengue virus

Xiang Guo^1,2^, Pingying Teng^1,3^, Ziyao Li^1^, Liqiang Li^1,4^, Xiaohua Liu^1^, Xiaoqing Zhang^1^, Yuji Wang^1^, Minling Hu^1^, Wenwen Ren^1^, Shu Zeng^1^, Haiyang Chen^1^, Liu Ge^1^, Shihan Liu^1^, Zhiqiang Peng^5^, Jiufeng Sun^6^, Xin Zhang^5^, Lei Luo^7^, Jie Peng^8^, Benyun Shi^9^, Rangke Wu^10^, Jiming Liu^11^, Fuchun Zhang^12^, Xiao-Guang Chen^1^, Tong Chen^13*^, Xiaohong Zhou^1*^

^1^ Institute of Tropical Medicine, Department of Pathogen Biology, School of Public Health, Southern Medical University; Guangdong Provincial Key Laboratory of Tropical Disease Research; Key Laboratory of Infectious Diseases Research in South China, Ministry of Education, Guangzhou 510515, Guangdong, China

^2^ School of Basic Medical Sciences, Henan University, Kaifeng, China

^3^ Department of Parasitology, Guilin Medical University, Guilin, China

^4^ Department of Clinical Laboratory, The Third People’s Hospital of Shenzhen, Southern University of Science and Technology, National Clinical Research Center for Infectious Diseases, Guangdong Provincial Clinical Research Center for Infectious Diseases (Tuberculosis), Shenzhen Clinical Research Research Center for Tuberculosis, Shenzhen 518112, China

^5^ Guangdong Provincial Center for Disease Control and Prevention, Guangzhou 511430, China

^6^ Guangdong Provincial Institute of Public Health, Guangdong Provincial Center for Disease Control and Prevention, Guangzhou 511430, China,

^7^ Guangzhou Center for Disease Control and Prevention, Guangzhou 510440, China

^8^ Department of Infectious Disease, Nanfang Hospital, Southern Medical University, Guangzhou 510515, China

^9^ College of Computer and Information Engineering, Nanjing Tech University, Nanjing 211816, China

^10^ The School of Foreign Studies, Southern Medical University, Guangzhou 510515, China

^11^ Department of Computer Science, Hong Kong Baptist University, Hong Kong 999077, China

^12^ Guangzhou Medical Research Institute of Infectious Diseases, Infectious Disease Center, Guangzhou Eighth People’s Hospital, Guangzhou Medical University, Guangzhou 510440, China

^13^ State Key Laboratory for Quality Ensurance and Sustainable Use of Dao-di Herbs, National Resource Center for Chinese Materia Medica, China Academy of Chinese Medical Sciences, Beijing 100000, China

*Correspondence: daizhouxh@163.com (Xiaohong Zhou), chent@nrc.ac.cn (Tong Chen)

**SUPPLEMENTARY METHODS**

**Database construction, data collection and processing**

The GISDD database was meticulously constructed by defining seven types of data categories including data quality, strain isolation, sequence information, genotyping, epidemiology information, contributed journal and institution, and note. These categories comprised forty-four items such as isolation host, sequencing technology, collection date, contributing institutes, etc. The database was also integrated with publicly accessible scientific databases like ViPR, National Microbiology Data Center (NMDC), and NCBI, with data duplication performed across these databases. The database encompassed articles and contributors' information related to these data items. A rigorous selection process starting from title and abstract screening down to full-text evaluation based on set inclusion and exclusion criteria was employed to identify eligible studies. Genotyping information details involving genotype, subgenotype, and clade affiliation for the included sequences was designated under the well-established global genotyping framework of dengue virus (DENV) [1]. The sequences are classified and labeled based on their length, gene integrity, and other relevant factors. In the GISDD database, a comprehensive classification system consisting of four levels is employed, which is elaborated in Table S1.

The data updating process in GISDD involves batch retrieval of sequences from public databases and repositories, followed by manual search for associated articles or data. The challenge lies in meticulously searching and confirming the association information of these sequences, which serves as a prominent feature of GISDD's dataset. With a proficient team dedicated to such updates, we have been consistently enhancing the data since the inception of this project, currently at version 1.3.2. We have provided supplementary logs on previous version updates and detailed information about our data updating process in Table S2.

**Website architecture**

The GISDD platform was implemented as a web application using Javascript and HTML for front-end development. The core JavaScript libraries used include Vue.js (https://vuejs.org) for the main frame, echarts (https://echarts.apache.org), plotly.js (https://plotly.com/) and D3.js (https://d3js.org/) for interactive charts. The backend data transporting was conducted using the high-level web framework Django (https://www.djangoproject.com). The Mysql open-source data management system is utilized for saving and accessing table data. Extensive browser compatibility testing has been conducted on various web browsers including Google Chrome, Firefox, and Internet Explorer, ensure a seamless user experience.

**GISDDprimer collecting published DENV primers and evaluating new designed ones**

The GISDDprimer provides access to a curated collection of published primer pairs/sets for PCR, qPCR, Cas detection, with available sequence information. For literature search strategy, we utilized specific terms such as ‘dengue’ OR ‘DENV’ AND (‘primer’ OR ‘detection’), as well as Chinese equivalents ‘登革热’ OR ‘登革病毒’ AND (‘引物’ OR ‘检测’), to search targeted English and Chinese databases like PubMed (https://www.ncbi.nlm.nih.gov/pubmed/), China National Knowledge Infrastructure Databases (CNKI, http://www.cnki.net), and Wanfang (<http://g.wanfangdata.com.cn/>) from inception to February 6th, 2024. To validate the amplification region and assess possible off-target effects, we designed a primer evaluation process for both collected primers and newly submitted primers based on various evaluation indices. These indices included the primer’s position within DENV genome, primer length, targeted serotype and fragment size specificity, and the potential off-target ratio. We also considered all DENV sequences and identified subgenotypes associated with any potential off-target sequences.

**GISDDref for DENV molecular epidemiology study**

For the DENV molecular epidemiology study, we conducted a literature search in English and Chinese databases using the same retrieval methods as described for GISDDprimer. The search included terms like ‘dengue’ OR ‘DENV’ as well as Chinese counterparts ‘登革热’ or ‘登革病毒’ and was concluded on February 6th, 2024. The literature review followed specific selection criteria, involving independent searches by authors, resolution of conflicts through expert consultation, and a meticulous screening process based on title, abstract, and full-text to exclude irrelevant studies. Additionally, other relevant records identified through references of selected reports or expert recommendations were included to ensure that all eligible studies concerning epidemiology or molecular epidemiology of DENV, including case reports, case series, reviews, cross-sectional and cohort studies, were included for descriptive analyses.

**GISDDrlearn for DENV genotyping**

Developed to enhance the classification of DENV genotypes, the GISDDrlearn employed a random forest classification algorithm to predict serotype, genotype, subgenotype, and clade of DENV genotypes. We randomly selected 60% of the sequences data for model training. The model was crafted using the R language with the ‘caret’ [2] (version 6.094) and ‘randomForest’ [3] (version 4.71.1) R-package, employing the optimal model (parameters of mtry and splitrule) were selected based on the value of accuracy. The left 40% data were selected for model validation. The GISDDrlearn model’s performance was validated through confusion matrix for each sequence's serotype, genotype and subgenotype predictions in the training set (Figure S4, Table S3). The trained GISDDrlearn model along with header files, metadata and detailed notes on genotype, subgenotype, and clade, are accessible on the GISSDrlearn GitHub repository (https://github.com/GuoXiang9399/GISDDrlearn-training).

**SIR scoring system for risk estimation**

Following genotyping of user-submitted sequences by the SIR workflow, risk estimation is conducted based on sequence isolation time, location, and other relevant metadata. This assessment employs a fully standardized scoring system, in which scores for each item are assigned to ensure cross-comparability. The scoring system comprises four item groups: "Global Epidemiology", "Pathogen Features", "Local Epidemiology", and "Transmission Sources", encompassing a total of 17 items. Each item is rated on a 5-point scale ranging from 1 to 5. Although the scoring criteria were initially developed subjectively by multiple authors through consensus, their robustness has been validated through consensus-building meetings, with decisions resolved by senior authors. Furthermore, the item scoring matrix will be updated annually based on the sequence data available in the GISDD. The final results are visualized using a wind rose chart, which provides a holistic overview of the original scores without aggregating total scores or establishing categorical grades. For instance, the "Global Epidemiology" item is graded based on the percentage of the subgenotype within the current global serotype distribution, categorized into five levels: "0-5%, 5-20%, 20-40%, 40-60%, and 60-100%."

**REFERENCES**

1. Li Liqiang, Guo Xiang, Zhang Xiaoqing, Zhao Lingzhai, Li Li, Wang Yuji, Xie Tian, et al. 2022. “A unified global genotyping framework of dengue virus serotype-1 for a stratified coordinated surveillance strategy of dengue epidemics.” Infectious Dissease of Poverty 11: 107. https://doi.org/10.1186/s40249-022-01024-5.

2. Kuhn Max. 2008. “Building Predictive Models in R Using the caret Package”. Journal of Statistical Software 5:1-26. https://DOI 10.18637/jss.v028.i05.

3. Breiman Leo. 2001. “Random Forests”. Machine Learning. 1:5-32. <https://doi.org/10.1023/A:1010933404324>.

**Supplementary figure**


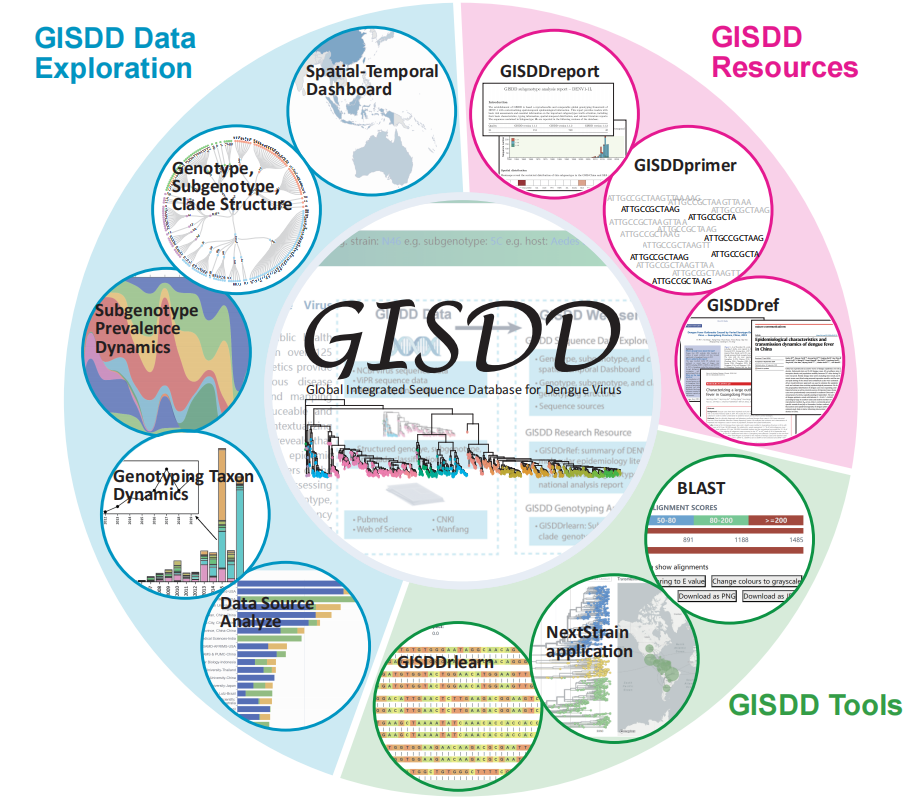


**Figure S1: Overview of GISDD data sources, web logical architecture, content.**


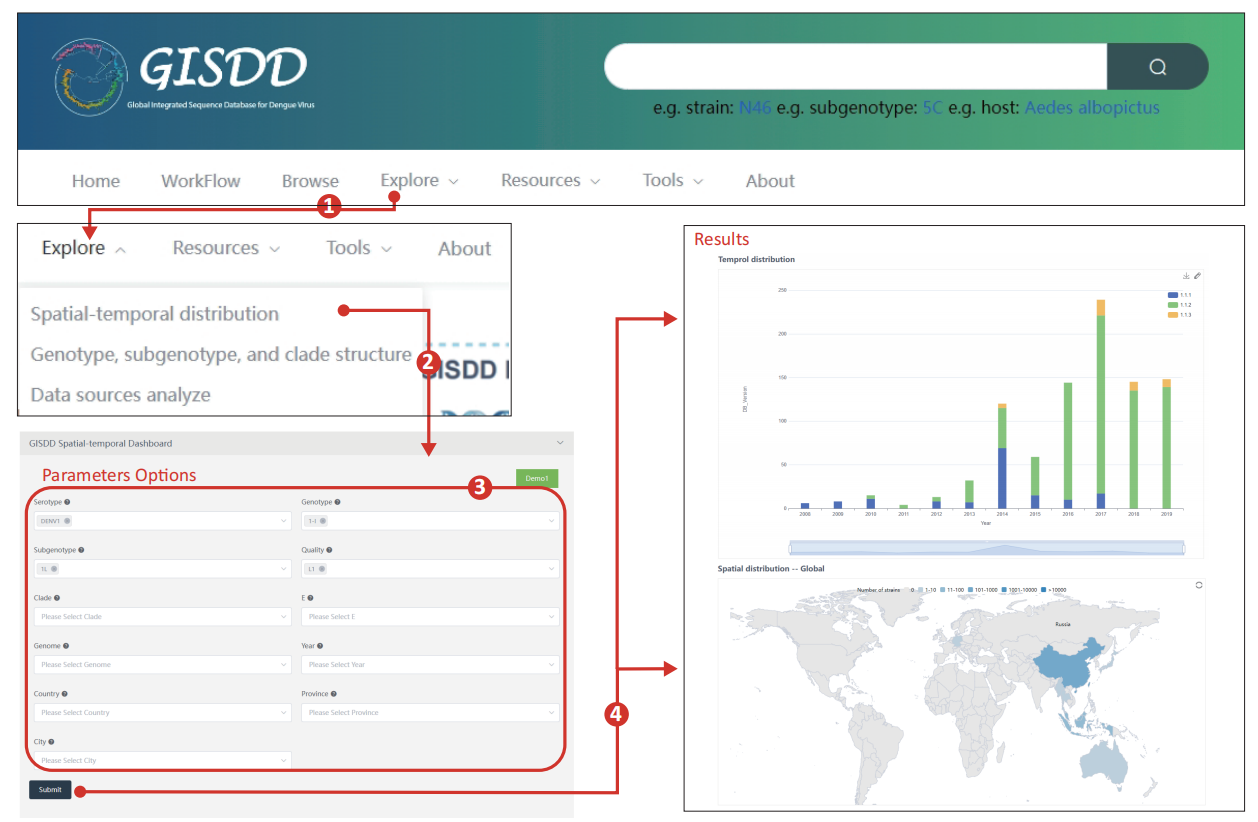


**Figure S2: The user interface of data exploration in GISDD.** Step 1: Click the ‘Explore’ button; Step 2: Select the first item ‘Spatial-temporal distribution’; Step 3: Select the parameters options, ‘Subgenotype DENV1, Genotype 1-I, Subgenotype 1L, Quality L1’ as a demo; Step 4: Check the results.


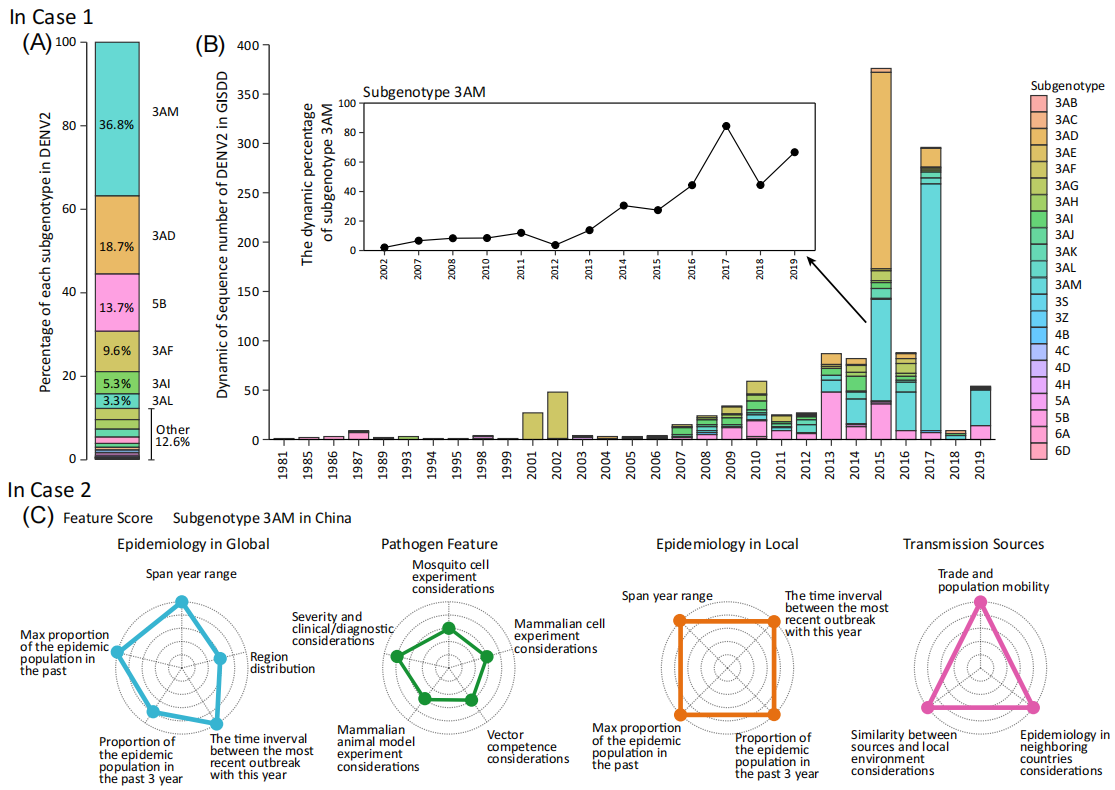


**Figure S3: SIR workflow implementation two cases.** (A) The DENV-2 subgenotype distribution chart indicates that the 3AM subgenotype constitutes a high proportion of the reported sequences in China. (B) Dynamics of subgenotype proportion among DENV-2 sequence counts in China. (C) Feature score result for SIR analysis for a local DENV-2 subgenotype 3AM epidemic in Shenzhen, China in 2024.


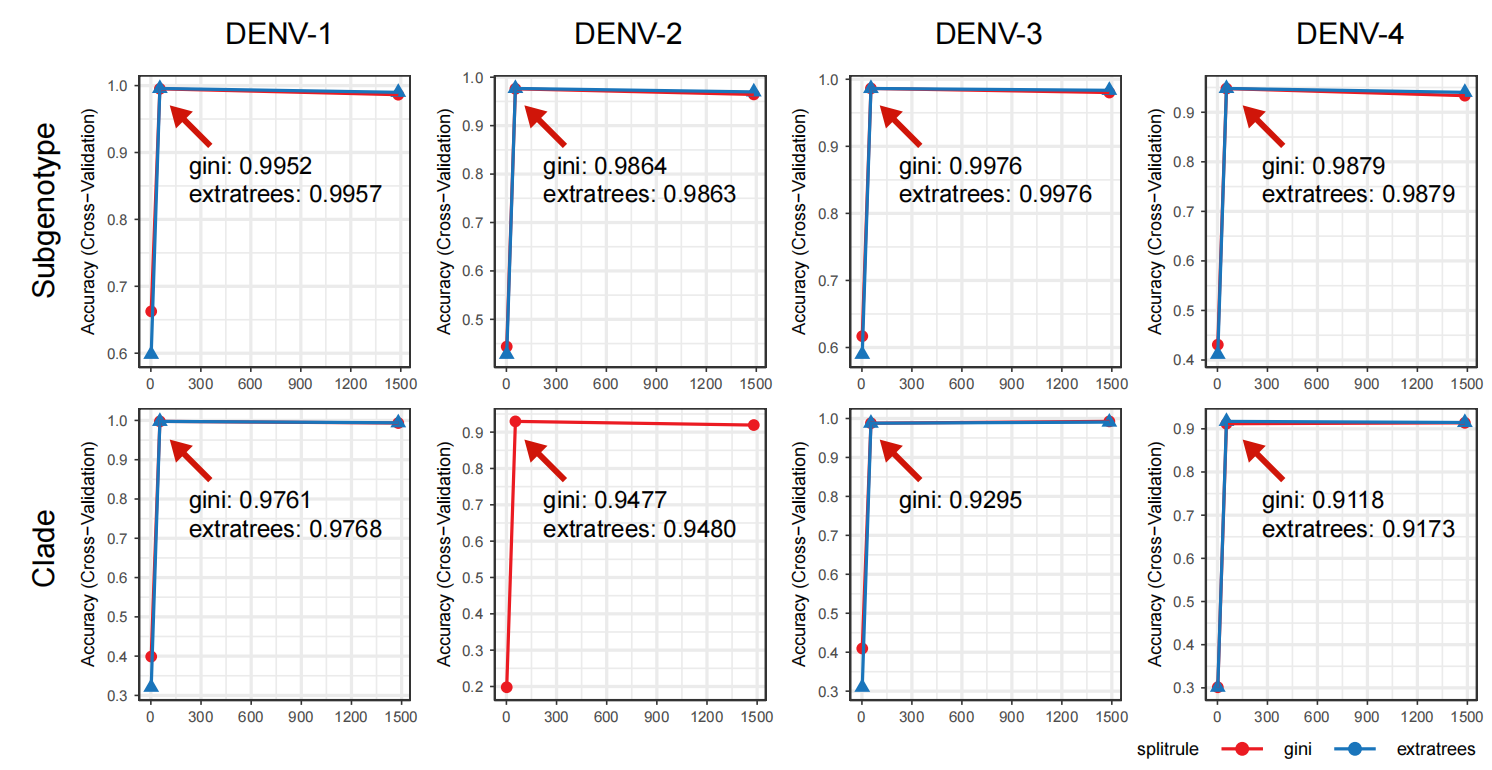


**Figure S4. Parameter optimization and classification effect of GISDDrlearn.** The GISDDrlearn model was crafted using the R language with the ‘caret’ (version 6.094) and ‘randomForest’ (version 4.71.1) R-package, employing the optimal model (parameters of mtry and splitrule) were selected based on the value of accuracy.
